# Artificial Neural Network Analysis-Based Immune-Related Signatures of Primary Non-Response to Infliximab in Patients With Ulcerative Colitis

**DOI:** 10.3389/fimmu.2021.742080

**Published:** 2021-12-21

**Authors:** Xuanfu Chen, Lingjuan Jiang, Wei Han, Xiaoyin Bai, Gechong Ruan, Mingyue Guo, Runing Zhou, Haozheng Liang, Hong Yang, Jiaming Qian

**Affiliations:** ^1^ Department of Gastroenterology, Peking Union Medical College Hospital, Peking Union Medical College, Chinese Academy of Medical Sciences, Beijing, China; ^2^ Medical Research Center, Peking Union Medical College Hospital, Peking Union Medical College, Chinese Academy of Medical Sciences, Beijing, China; ^3^ Department of Epidemiology and Biostatistics, Institute of Basic Medical Sciences, Chinese Academy of Medical Sciences and School of Basic Medicine, Peking Union Medical College, Beijing, China

**Keywords:** infliximab, ulcerative colitis, artificial neural network analysis, prediction, primary non-response

## Abstract

Infliximab (IFX) is an effective medication for ulcerative colitis (UC) patients. However, one-third of UC patients show primary non-response (PNR) to IFX. Our study analyzed three Gene Expression Omnibus (GEO) datasets and used the RobustRankAggreg (RRA) algorithm to assist in identifying differentially expressed genes (DEGs) between IFX responders and non-responders. Then, an artificial intelligence (AI) technology, artificial neural network (ANN) analysis, was applied to validate the predictive value of the selected genes. The results showed that the combination of *CDX2*, *CHP2*, *HSD11B2*, *RANK*, *NOX4*, and *VDR* is a good predictor of patients’ response to IFX therapy. The range of repeated overall area under the receiver-operating characteristic curve (AUC) was 0.850 ± 0.103. Moreover, we used an independent GEO dataset to further verify the value of the six DEGs in predicting PNR to IFX, which has a range of overall AUC of 0.759 ± 0.065. Since protein detection did not require fresh tissue and can avoid multiple biopsies, our study tried to discover whether the key information, analyzed by RNA levels, is suitable for protein detection. Therefore, immunohistochemistry (IHC) staining of colonic biopsy tissues from UC patients treated with IFX and a receiver-operating characteristic (ROC) analysis were used to further explore the clinical application value of the six DEGs at the protein level. The IHC staining of colon tissues from UC patients confirmed that VDR and RANK are significantly associated with IFX efficacy. Total IHC scores lower than 5 for VDR and lower than 7 for RANK had an AUC of 0.828 (95% CI: 0.665–0.991, *p* = 0.013) in predicting PNR to IFX. Collectively, we identified a predictive RNA model for PNR to IFX and explored an immune-related protein model based on the RNA model, including VDR and RANK, as a predictor of IFX non-response, and determined the cutoff value. The result showed a connection between the RNA and protein model, and both two models were available. However, the composite signature of VDR and RANK is more conducive to clinical application, which could be used to guide the preselection of patients who might benefit from pharmacological treatment in the future.

## Introduction

Ulcerative colitis (UC) is a chronic relapsing inflammatory disease of the colonic mucosa. UC is a relapsing disease requiring long-term management throughout life. The mainstay therapies for UC include 5-aminosalicylates, glucocorticoids, immunosuppressants, and biologic agents (1, 2). Biologic drugs, including antitumor necrosis factor (TNF)-α agents, anti-integrin drugs (vedolizumab), Janus kinase inhibitors (tofacitinib), and interleukin-12/23 antibodies (ustekinumab) (3), have driven UC therapy to a new era. The anti-TNF-α agent infliximab (IFX) is the oldest and most widely used biologic agent.

A meta-analysis showed that IFX was the highest-ranking biologic agent for the induction of clinical remission (OR 4.10, 95% CI: 2.58–6.52) and mucosal healing in moderate to severe UC (4, 5). However, according to previous studies, nearly one-third of UC patients show primary non-response (PNR). Moreover, studies have shown that other biologic agents have a higher failure rate in patients who previously failed to respond to IFX treatment than in those who are naïve to anti-TNF treatment (6, 7). Furthermore, the time PNR patients spend on IFX therapy can delay treatment, increase the risk of disease aggravation, and increase the economic burden of UC. Therefore, it is crucial to distinguish between PNR and effective responses to IFX treatment. Predictions of non-responses to IFX can assist the accurate selection of patients who could experience a clinical benefit and avoid potential adverse effects and unnecessary financial investment. Thus, an approach to identify markers from common, accessible samples, such as tissue biopsies or blood samples, is needed.

Previous studies have demonstrated that the therapeutic response depends on clinical factors, serum markers, and host genetics. Brandse et al. found that a high baseline serum level of C-reactive protein (CRP) was associated with lower serum concentrations of IFX, leading to non-response (8). Arias et al. identified a panel of serum markers (pANCA, CRP, and albumin) as independent predictors of the long-term outcome following IFX therapy in UC patients (9). Nevertheless, these indexes mainly related to disease activity and imperfectly predicted the primary therapeutic response to IFX (10). Burke et al. showed that genetic polymorphisms have predictive value for PNR to anti-TNF therapy in UC patients (11). Moreover, a high pretreatment expression of oncostatin M (OSM) was associated with anti-TNF resistance (12). However, the signatures of anti-TNF non-response mentioned above need further external clinical validation.

In the present study, we aimed to identify the specific markers underlying the PNR to IFX using combined datasets. Due to the expense of RNA sequencing, the RNA-seq dataset was small, and we used the bootstrapping method to randomly resample (13, 14). The first step to developing a predictor for clinical application is to find a repeatable result. We used an artificial intelligence (AI) technology, artificial neural network (ANN) analysis, to validate whether these data might be useful in estimating PNR. We repeated this process multiple times to validate the results. Moreover, we used another independent RNA dataset to confirm the result. Furthermore, an immunohistochemistry staining of colon tissue from UC patients who underwent IFX therapy was performed to explore the clinical application at the protein level. Ultimately, the findings of this work provide a greater understanding of which patients might receive therapeutic benefit from IFX therapy.

## Methods

### Data Collection From the Gene Expression Omnibus Database

This study acquired clinical data and mRNA expression profiles of colon tissue from adult patients with UC from the Gene Expression Omnibus (GEO) database (https://www.ncbi.nlm.nih.gov/geo/). By using the keywords “ulcerative colitis” or “UC” and “IFX” or “infliximab,” a total of eight series associated with UC treated by IFX were identified. After review, we selected three datasets (GSE12251, GSE16879, and GSE23597) containing the therapeutic efficacy of different dosages of IFX (15–18) as a discovery cohort. The platform used for the three datasets was the GPL570 [HG-U133_Plus_2] Affymetrix Human Genome U133 Plus 2.0 Array. The selected patients all underwent colonoscopy, and biopsies of the diseased colon were performed before IFX therapy. Since the most commonly used dose of IFX in the clinic is 5 mg/kg and to maintain consistency among the three datasets, we selected patients who received a 5-mg/kg dose of IFX from the three datasets. Finally, 25 UC patients who responded to the first IFX treatment and 25 UC patients who exhibited PNR were included. The response was assessed in week 8 in the GSE12251 and GSE23597 datasets after the first infliximab treatment, and in weeks 4–6 in the GSE16879 dataset. The response definition was complete mucosal healing with a Mayo endoscopic subscore of 0 or 1 and a histological score of 0 or 1. An independent cohort from GEO (GSE73661) was used for further validation, which contained eight primary IFX responders and 15 non-responders. The response was assessed in weeks 4–6 in the GSE73661 dataset. The platform of the GSE73661 dataset was the GPL6244 [HuGene-1_0-st].

### Data Extraction, Screening, and Aggregation of Differentially Expressed Genes

The pre-IFX-therapy-sequencing data of the obtained patients were extracted from the GSE12251, GSE16879, and GSE23597 datasets. UC patients who responded or did not respond to a 5-mg/kg dose of IFX at the first follow-up were selected and divided into the response group and PNR group. The limma R package (http://www.bioconductor.org/) was used to filter the Differentially Expressed Genes (DEGs) in each dataset. The same analysis was done in the validation cohort, the GSE73661 dataset. DEGs were defined as both an adjusted *p*-value < 0.05 and |log fold change (logFC)| > 0.5. The TXT files of all DEGs of the discovery datasets were sorted by logFC and saved for the subsequent integration analysis.

The three TXT files of all DEGs sorted by logFC were aggregated using the RobustRankAggreg (RRA) R package (https://CRAN.R-project.org/package=RobustRa-nkAggreg). The aggregated DEGs from all datasets, including upregulated and downregulated DEGs, were saved for subsequent analysis.

We selected aggregated upregulated and downregulated genes with a *p*-value lower than 0.05. Then, we ranked the genes by the logFC in order from the largest to the smallest. We reviewed the significant protein-coding DEGs and sorted out the genes expression in the alimentary tract through NCBI (https://www.ncbi.nlm.nih.gov/). We then reviewed published papers to determine genes which associate with immune activities to construct a list of proteins linked to the efficacy of IFX. Subsequently, we used the GSE16879 dataset, which contained sequencing data before and after IFX therapy, to determine the relationship between the selected protein-coding genes and IFX. Since the fewer indicators included, the higher the economic benefits obtained, we tried to find a better combination of DEGs.

### Resampling Method and Artificial Neural Network Analysis

The subjects in the response group and PNR group were resampled by the “bootstrap” method. The dataset was randomly resampled to 250 by the proportion of the two groups (with replacement, i.e., when an item is sampled, it is immediately returned) (13). The samples from the resampling were analyzed by an ANN to show the efficiency of the model. To confirm the stability of the model, we repeated the resampling and ANN analysis 500 times. The process was also performed by shielding one input randomly. The range of area under the receiver-operating characteristic curve (AUC) was calculated. The same analysis parameters of ANN were used to verify the prediction ability of the selected DEGs in the validation dataset.

### Exploring the Expression of the Selected DEGs at the Protein Level

Patients with UC receiving IFX monotherapy were enrolled from 2017 to 2020 at the Peking Union Medical College Hospital (PUMCH). Twenty-four UC patients were selected. The diagnostic criteria were based on the third European Crohn’s and Colitis Organization (ECCO) consensus guideline for UC and the 2018 Chinese consensus for inflammatory bowel disease (19, 20). We evaluated their clinical data at baseline, week 6, and week 14 after therapy. The response to IFX in week 6 was defined as a decrease in the partial Mayo score (Mayo score without endoscopy) of at least three points and at least 30% compared with the baseline data (21). A response in week 14 was defined as a decrease in the Mayo score of at least three points and at least 30% less than the baseline value, and the rectal bleeding score should decrease by more than 1 point or be equal to 0 or 1 point. The colonoscopic biopsies before the first IFX treatment of these patients were used for the immunohistochemistry (IHC) staining to verify the effectiveness of the obtained genes at the protein level. All colonic biopsy samples and clinical data of the patients used in this study were carried out with the approval of the Peking Union Medical College Hospital and the Chinese Academy Medical Science Ethics Committee (S-K1142).

### Staining Off Target Proteins by Immunohistochemistry

We performed IHC staining off of the target proteins in formalin-fixed, paraffin-embedded colon tissues. The antigens were retrieved by boiling the samples for 10 min in 10 mM citrate (pH 6.0) or EDTA antigen repair solution (pH 9.0) (ZSGB-BIO). The slides were stained with rabbit monoclonal antibodies (Cell Signaling) and then incubated with a peroxidase-conjugated secondary antibody. Finally, the signals were visualized with diaminobenzidine (DAB) peroxidase substrate kit (Servicebio).

### IHC Scoring

**Figure 1:**
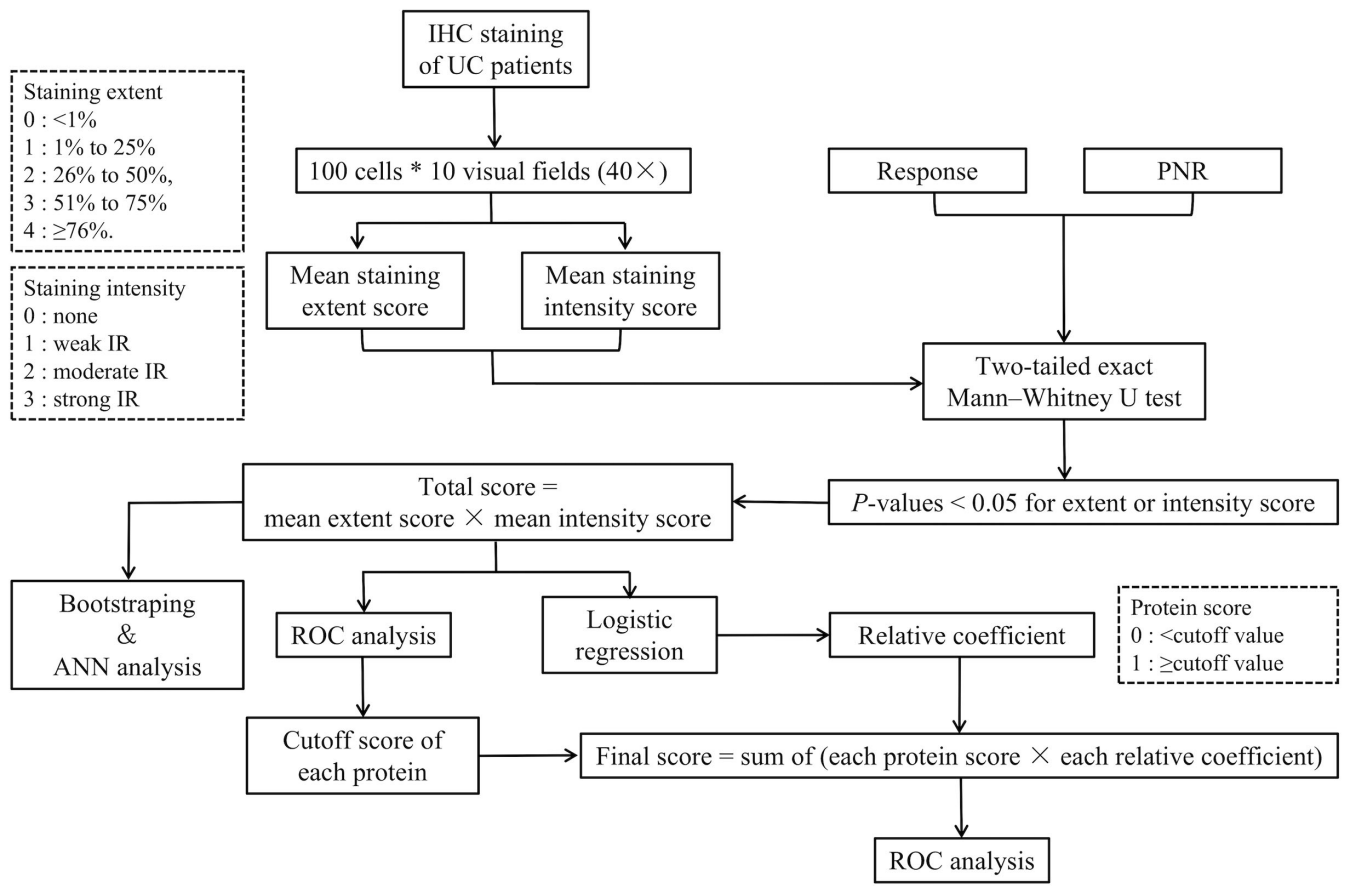
Flowchart of the IHC scoring and analysis. IHC, immunohistochemistry; PNR, primary non-response; ANN analysis, artificial neural network analysis; ROC, receiver-operating characteristic.

A flowchart of the IHC scoring and analysis is shown in **Figure 1**. The IHC staining was semiquantitatively evaluated by rating both the extent and intensity. First, we randomly selected 10 visual fields (×40) under a light microscope and counted 100 cells in each visual field. Then, we rated the extent as the proportion of positive cells on a scale from 0 to 4 as follows: 0, <1%; 1, 1% to 25%; 2, 26% to 50%; 3, 51% to 75%; and 4, ≥76%. Moreover, the intensity of the immunoreactivity (IR) was rated on a scale from 0 to 3 as follows: 0, no IR; 1, weak IR; 2, moderate IR; and 3, strong IR (**Table 1**) (22). We defined the IHC score of each protein as the mean value of the extent or intensity score in each visual field. The IHC scoring was analyzed independently by two gastroenterologists who were blinded to the patients’ response to IFX.

**Table 1:** Grading scale for the semiquantitative IHC scoring.

Score	Staining extent	Staining intensity
0	<1%	None
1	1%–25%	Weak immunoreactivity
2	26%–50%	Moderate immunoreactivity
3	51%–75%	Strong immunoreactivity
4	≥76%	–

Additionally, we used a two-tailed exact Mann–Whitney U test (non-parametric) to compare the IHC score between the responders and non-responders. Any variable with a *p*-values lower than 0.05 in its extent or intensity scores was included in the multivariate analysis. Then, we defined the *total score* of each subject as the product of the mean extent and intensity score of each protein as follows:


Total IHC score of each protein in each sample = mean extent score×mean intensity score

Moreover, we used the bootstrap method and an ANN analysis to show the efficiency of the combination of the selected proteins in predicting IFX efficacy. The resampling and ANN analysis process were repeated 500 times. The range of AUCs was calculated to measure the results of ANN analysis of the proteins in predicting the therapeutic effect of IFX in week 6 and week 14.

To achieve the threshold of distinction between a response and PNR, we divided the cutoff value of the total IHC score of each included protein by an ROC analysis. Moreover, the *protein score* was defined as 1 when the total IHC score was greater than or equal to the cutoff value and 0 when the total IHC score was lower than the cutoff value. Then, we used a logistic regression to calculate the relative coefficient of the IHC score of the proteins. We divided the regression coefficient of the other variables by the minimum regression coefficient and rounded the result to obtain the score of each variable. The product of the relative coefficient and protein score was obtained, and the sum of the products was defined as the final predictive score. An ROC curve was plotted to estimate the value of the selected proteins in predicting the therapeutic effect of IFX.


Final score = protein score 1 × relative coefficient 1 + protein score 2 × relative coefficient 2+…+protein score n × relative coefficient n

### Statistical Analysis

Non-parametric analyses were used to estimate the differences between the IFX response and non-response groups. The statistical tests were two-tailed and described in the figure legends. ROC curves were used to test the prediction value. All *p*-values less than 0.05 were considered significant. All analyses and the graph creation were performed in SPSS (version 25.0, IBM Corporation, Chicago, USA), R software (version 3.5.2, R Foundation for Statistical Computing, Vienna, Austria), and MATLAB (R2019a, MathWorks, USA).

## Results

### Identification of DEGs Between Responders and Primary Non-Responders

**Figure 2:**
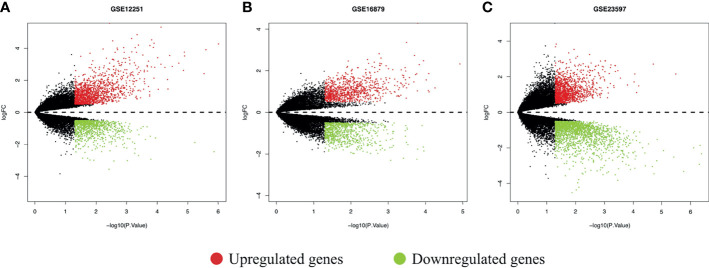
DEGs between responders and non-responders in each dataset shown in volcano plots. **(A)** Volcano plot of the GSE12251 dataset; **(B)** volcano plot of the GSE16879 dataset; and **(C)** volcano plot of the GSE23597 dataset. The red dots represented upregulated genes based on a *p*-value < 0.05 and logFC > 0.5; the green dots represented downregulated genes based on a *p*-value < 0.05 and logFC<-0.5; the black spots represented genes with no significant difference in expression. DEGs, differentially expressed genes; logFC, log-fold change.

According to the inclusion criteria for the sequencing data before 5 mg/kg IFX therapy, we extracted UC patients who were primary IFX responders or non-responders from the GSE12251, GSE16879, and GSE23597 datasets. The GSE12251 dataset included four responders and seven non-responders, the GSE16879 dataset contained eight responders and 16 non-responders, and the GSE23597 dataset included 13 responders and two non-responders (**Table 2**). The DEGs were screened using the limma R package (adjusted *p*-value < 0.05 and |logFC| > 0.5). The GSE12251 dataset contained 2,335 DEGs, including 1,346 upregulated genes and 989 downregulated genes. Furthermore, 934 upregulated genes and 852 downregulated genes were included in the GSE16879 dataset, resulting in a total of 1,786 DEGs in this dataset. Finally, the GSE23597 dataset contained 3,497 DEGs, including 1,390 upregulated genes and 2,107 downregulated genes. The DEGs in the three datasets are shown in **Table 3** and **Figure 2**.

**Table 2:** Details of the UC patients receiving IFX therapy in the GEO database.

GEO dataset	Platform	PubMed ID	Sample	Time of biopsy	Time of assessment	Response	PNR
GSE12251	GPL570	19700435	Colonic tissue	Within 2 weeks before treatment	Week 8	4	7
GSE16879	GPL570	19956723	Colonic tissue	Within 1 week before treatment	Weeks 4–6	8	16
GSE23597	GPL570	21448149, 31039157	Colonic tissue	Within 2 weeks before treatment	Week 8	13	2

PNR, primary non-response.

**Table 3:** DEGs between the responders and non-responders in each dataset.

	Upregulated genes (*p*-value < 0.05 and logFC > 0.5)	Downregulated genes (*p*-value < 0.05 and logFC < -0.5)
GSE12251	1346	989
GSE16879	934	852
GSE23597	1390	2107

### Integrated DEGs Between Responders and Primary Non-Responders

**Figure 3:**
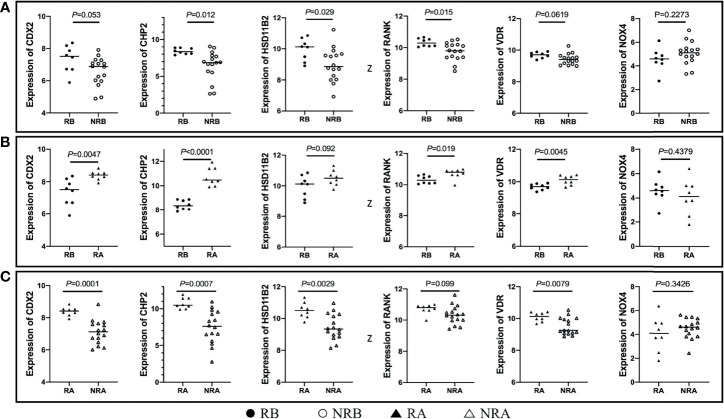
DEG expression in different stages of IFX treatment. **(A)** The non-responders of IFX tended to have a lower pretreatment expression of the downregulated DEGs compared with the responders, and NOX4 displayed the opposite results; **(B)** the posttreatment expression of the downregulated DEGs exhibited a trend of increases in the responders, and NOX4 exhibited the opposite results; **(C)** the expression of the downregulated DEGs after treatment in those who responded to IFX was higher than that those who did not respond to IFX, and NOX4 exhibited the opposite results. DEGs, differentially expressed genes; IFX, infliximab; RB, sequencing data of responders before IFX therapy; NRB, sequencing data of non-responders before IFX therapy; RA, sequencing data of responders after IFX therapy; NRA, sequencing data of non-responders after IFX therapy.

**Figure 4:**
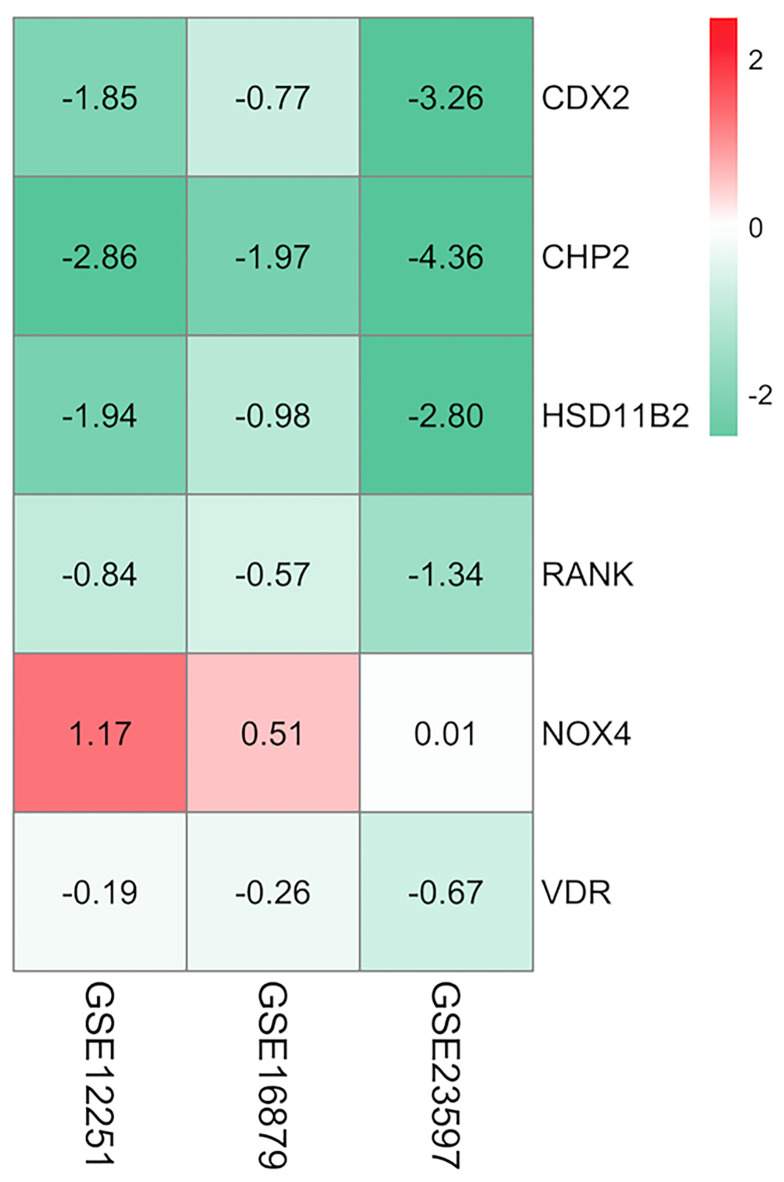
Heatmap of the selected proteins. CDX2, CHP2, HSD11B2, RANK, and VDR displayed decreased expression, while NOX4 displayed increased expression; the red color represented logFC > 0, the green color represented logFC < 0 and the value in the box represented the logFC value. logFC, log fold change.

The aggregated DEGs were screened by the RRA package (*p*-value < 0.05, |logFC| > 0.5). This method was based on the RRA algorithm in which each gene in each dataset was randomly arranged. If a gene ranked higher in all datasets, the associated *p*-value was lower, indicating that the possibility of this gene being a DEG was greater in all datasets. Using the RRA method, 624 integrated DEGs were identified, consisting of 18 upregulated genes and 606 downregulated genes. We selected the aggregated upregulated and downregulated DEGs by an associated *p*-value lower than 0.05, ranked the logFC in order from the largest to the smallest, identified the protein-coding genes, and determined the gene expression in the gastrointestinal tract by NCBI. Among the upregulated genes, those with low expression in the normal gastrointestinal tract were selected, while among the downregulated genes, those with high expression in the normal gastrointestinal tract were selected. We then reviewed published papers to consider proteins linked to immune or inflammatory processes. Ultimately, five downregulated proteins associated with PNR, including CDX2, CHP2, HSD11B2, RANK, and VDR, were selected; one upregulated protein, NOX4, was chosen. Furthermore, we used the GSE16879 dataset, which contained RNA sequencing data both before and after IFX treatment, to determine the relationship between the selected DEGs and IFX therapy. We found that 1) the non-responders to IFX tended to have a lower pretreatment expression of the downregulated DEGs compared with the responders; 2) the posttreatment expression of the downregulated DEGs displayed a trend of increases in the responders; and 3) the expression of the downregulated DEGs after treatment in those who responded to IFX was higher than that in those who did not respond to IFX. This phenomenon was the opposite in the upregulated DEG NOX4 (**Figure 3** **)**. Thus, the following six proteins were ultimately selected for the construction of the predictive model of IFX efficacy: CDX2, CHP2, HSD11B2, RANK, NOX4, and VDR (**Figure 4**). CDX2, CHP2, HSD11B2, RANK, and VDR showed decreased expression in the non-responders, while NOX4 showed increased expression.

### Resampling and ANN Analysis Results of the DEGs in the Discovery and Validation Cohort

We used the bootstrap method to randomly resample the response group (n = 25) and PNR group (n = 25) and enlarge the sample size to 250 in proportion of the two groups. Bootstrapping can reduce heterogeneity in different sample populations and avoid the problem of sample reduction caused by cross validation. Then, we used the resampled dataset to perform an ANN analysis (23, 24). The ANN analysis weighed the importance of the selected proteins, thus predicting the effect on achieving response to IFX therapy. Based on the collection of connected units, ANN loosely mimics neurons in the real brain. Each connection works as synapses in a biological brain. ANN can convey signals from one artificial neuron to another. Then, artificial neurons that receive signals can transmit these signals and signal additional artificial neurons connected to them. In typical ANN applications, the signals at a connection between artificial neurons are actual numbers and the outputs of each artificial neuron are calculated by a non-linear function of the sum of its inputs. Artificial neurons and their connections have a weight that adjusts as learning proceeds. The weight enhances or reduces the power of the signals at a connection in the ANN. ANN incorporates a system of interconnections based on simple mathematical models associated with learning algorithms. ANN consists of a four-layer (one input layer, two hidden layers, and one output layer) feedforward analysis. To develop the ANN, cases were randomly assigned to a training set (70%), test set (15%), and verification set (15%) through a generator of random numbers in our study. Backpropagation of error was applied as a learning rule by the online training method. The synaptic weights were calculated after each training data record.

**Figure 5:**
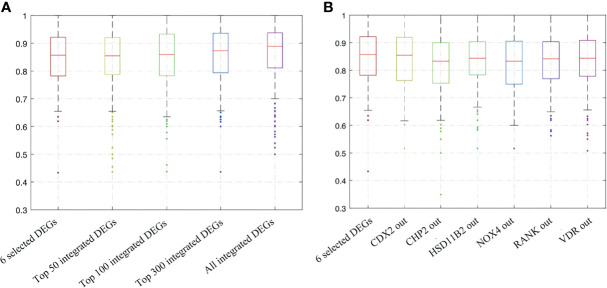
Bootstrapping and ANN analysis results of the top DEGs, the six selected DEGs, and shielding of one DEG randomly based on the latter. **(A)** Analysis results of all integrated DEGs, top 300, top 100, top 50, and the six selected DEGs; **(B)** analysis results of shielding of one input randomly based on the six selected DEGs. ANN analysis, artificial neural network analysis; DEGs, differentially expressed genes.

As the more included the indicators, the higher economic burden for application, we tried different combinations of DEGs to find a better small protein combination. We performed the resampling and ANN analysis 500 times by selecting all integrated DEGs, top 300, top 100, top 50, and the six selected DEGs. The process was also performed by shielding one input randomly based on the six selected DEGs. The range of the repeated overall AUC of the six selected DEGs was 0.850 ± 0.103, which was similar to the different combination of the top DEGs (**Figure 5A** and **Supplementary Table 1** **)** and was slightly higher than that of the shielding-one-DEG model based on the six selected DEGs (**Figure 5B** and **Table 4**). The results showed that the six-DEG model had good economic benefits and performed better in predicting the IFX response. The repeated results demonstrated that the model was stable. We also performed an ANN analysis in the independent GEO dataset (GSE73661). The results showed that the range of repeated overall AUC was 0.759 ± 0.065, indicating that the model was feasible.

**Table 4:** AUC of different combinations of the six selected DEGs.

DEGs combination	AUC (mean ± SD)
Six selected DEGs	0.850 ± 0.103
CDX2_out	0.837 ± 0.106
CHP2_out	0.823 ± 0.115
HSD11B2_out	0.833 ± 0.100
NOX4_out	0.829 ± 0.105
RANK_out	0.836 ± 0.100
VDR_out	0.838 ± 0.103

### Exploring Results of IHC in UC Patients Undergoing IFX Therapy

**Figure 6:**
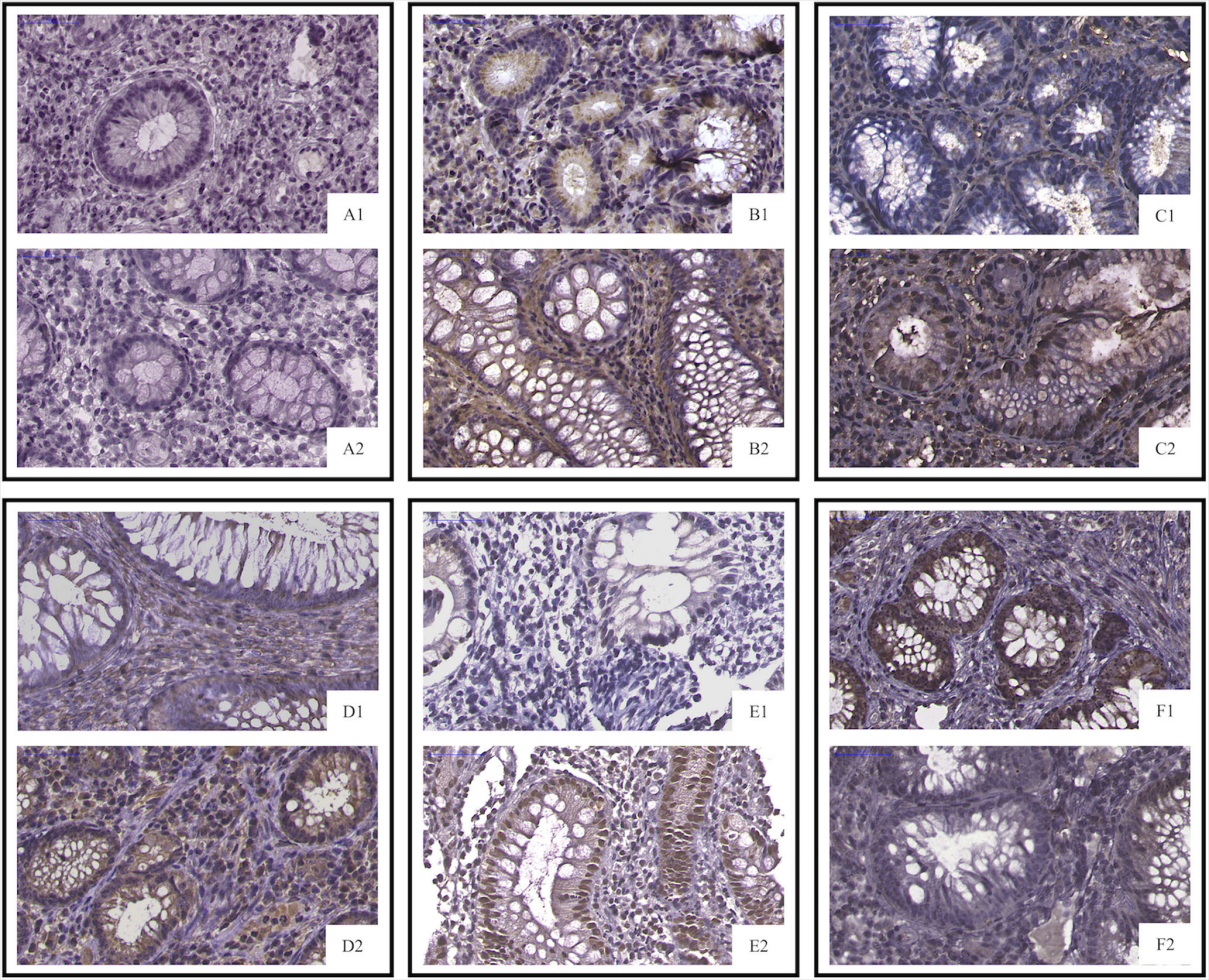
IHC staining of selected proteins. (**A1**, PNR; **A2**, response) CDX2 did not differ between the primary IFX non-responders and responders; (**B1**, PNR; **B2,** response) CHP2 (**C1**, PNR; **C2**, response), HSD11B2 (**D1**, PNR; **D2**, response), RANK (**E1**, PNR; **E2**, response), and VDR staining was decreased in the primary non-responders, while NOX4 (**F1**, PNR; **F2**, response) was increased in the non-responders. PNR, primary non-response.

Biopsies are usually taken for pathological examination when UC patients undergo colonoscopy in the clinic. IHC analysis of clinical residual paraffin sections can avoid multiple biopsies and reduce the examination cost and time of patients. Thus, we tried to discover whether the key information, analyzed by RNA levels, is suitable for protein level detection. We used IHC analysis to explore the protein expression based on the selected DEGs and find clinical application predictors. Twenty-four UC patients were recruited from 2017 to 2020 at the Peking Union Medical College Hospital. Among these patients, 70.8% (n = 17) clinically responded to IFX treatment by week 6, and 29.2% (n = 7) did not. In addition, 54.17% (n = 13) of the patients achieved therapeutic benefits by week 14, while 45.83% (n = 11) did not. The proteins predicting IFX efficacy were evaluated by IHC scoring (**Figure 6**) without knowledge of the clinical data. After the analysis, CHP2, HSD11B2, RANK, and VDR were found to have reduced mean IHC extent and intensity scores in the non-response group, and NOX4 had increased scores, which is consistent with the results of the analysis of the GEO datasets, while CDX2 had a limited difference between the groups. VDR and RANK statistically significantly differed between the two groups in terms of the intensity scores (*p*-value <0.05), and VDR showed a trend-level difference in terms of the extent scores (*p*-value = 0.065) (**Tables 5**, **6**). These two proteins were selected for further analysis.

**Table 5:** The extent of the staining of IFX efficacy-predicting proteins in colonic biopsies from UC patients.

Variable	Staining extent score					Mean score	*p*-value*^a^*
	0 (n, %)	1 (n, %)	2 (n, %)	3 (n, %)	4 (n, %)		
CDX2							0.757
Responders	13 (76.5%)	4 (23.5%)	0	0	0	0.24	
Non-responders	5 (71.4%)	1 (14.3%)	1 (14.3%)	0	0	0.34	
HSD11B2							0.534
Responders	1 (5.9%)	6 (35.3%)	5 (29.4%)	4 (23.5%)	1 (5.9%)	1.88	
Non-responders	0	4 (57.1%)	2 (28.6%)	1 (14.3%)	0	1.57	
CHP2							0.209
Responders	0	3 (17.6%)	4 (23.5%)	5 (29.4%)	5 (29.4%)	2.71	
Non-responders	0	0	6 (85.7%)	1 (14.3%)	0	2.14	
RANK							0.114
Responders	0	0	6 (35.3%)	5 (29.4%)	6 (35.3%)	3.00	
Non-responders	0	1 (14.3%)	3 (42.9%)	3 (42.9%)	0	2.29	
NOX4							0.234
Responders	0	0	12 (70.6%)	5 (29.4%)	0	2.29	
Non-responders	0	0	3 (42.9%)	3 (42.9%)	1 (14.3%)	2.71	
VDR							0.065
Responders	0	5 (29.4%)	8 (47.1%)	3 (17.6%)	1 (5.9%)	2.00	
Non-responders	0	5 (71.4%)	2 (28.6%)	0	0	1.29	

^a^A Mann–Whitney U test was used for the analysis.

**Table 6:** The intensity of the staining of IFX efficacy-predicting proteins in colonic biopsies from UC patients.

Variable	Staining intensity score				Mean score	*p*-value*^a^*
	0 (n, %)	1 (n, %)	2 (n, %)	3 (n, %)		
CDX2						0.757
Responders	13 (76.5%)	4 (23.5%)	0	0	0.24	
Non-responders	5 (71.4%)	1 (14.3%)	1 (14.3%)	0	0.43	
HSD11B2						0.166
Responders	1 (5.9%)	5 (29.4%)	8 (47.1%)	3 (17.6%)	1.76	
Non-responders	0	5 (71.4%)	2 (28.6%)	0	1.29	
CHP2						0.455
Responders	0	0	10 (58.8%)	7 (41.2%)	2.41	
Non-responders	0	1 (14.3%)	4 (57.1%)	2 (28.6%)	2.14	
RANK						0.034
Responders	0	0	7 (41.2%)	10 (58.8%)	2.59	
Non-responders	0	2 (28.6%)	4 (57.1%)	1 (14.3%)	1.86	
NOX4						0.349
Responders	0	1 (5.9%)	8 (47.1%)	8 (47.1%)	2.41	
Non-responders	0	0	2 (28.6%)	5 (71.4%)	2.71	
VDR						0.024
Responders	0	0	2 (11.8%)	15 (88.2%)	2.88	
Non-responders	0	0	5 (71.4%)	2 (28.6%)	2.29	

^a^A Mann–Whitney U test was used for the analysis.

**Figure 7:**
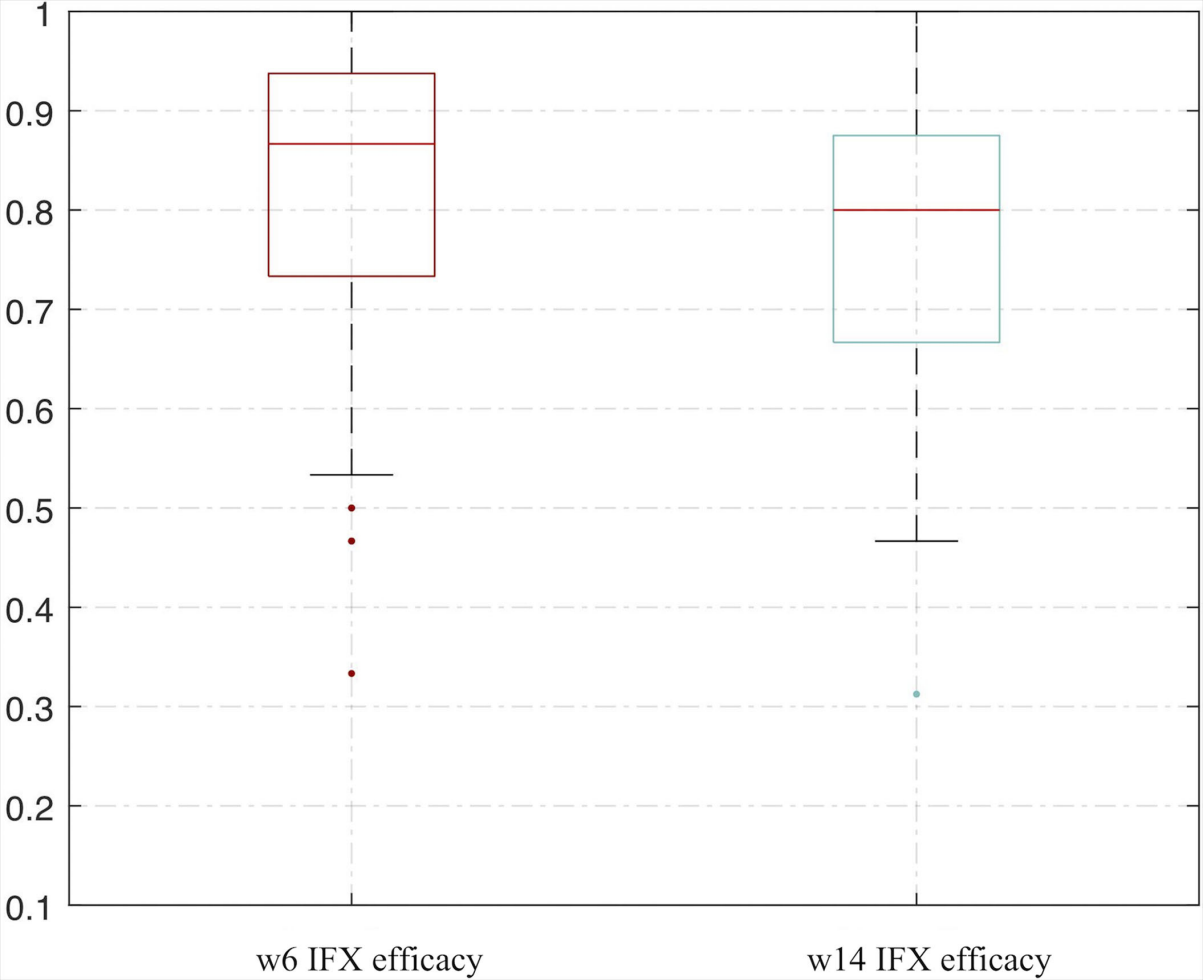
Bootstrapping and ANN analysis results of VDR and RANK in predicting IFX efficacy in week 6 and week 14. ANN analysis, artificial neural network analysis; IFX, infliximab.

We used the bootstrap method and an ANN analysis of VDR and RANK and repeated the analysis process 500 times. The AUC performed well in predicting the effect of IFX therapy. The range of repeated overall AUC was 0.837 ± 0.152 in predicting IFX efficacy in week 6 and was 0.776 ± 0.162 in predicting IFX efficacy in week 14 (**Figure 7** and **Supplementary Table 2** **)**.

To determine the cutoff values for VDR and RANK, we used an ROC analysis. Ultimately, the cutoff value of the total IHC score was 5 for VDR and 7 for RANK. In addition, the logistic regression analysis showed that the regressive equation was as follows:


logit (P)= −0.799 (total IHC score of VDR) − 0.44 (total IHC score of RANK) + 5.024

**Figure 8:**
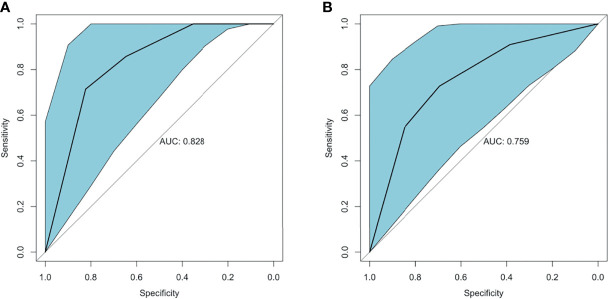
ROC curve of VDR and RANK in predicting IFX efficacy in week 6 and week 14. **(A)** The AUC of the estimation 6 weeks after IFX treatment was 0.828. **(B)** The AUC of the estimation 14 weeks after IFX treatment was 0.759. ROC, receiver-operating characteristic; AUC, area under the receiver-operating characteristic curve; IFX, infliximab.

Therefore, the relative coefficient of VDR was 2, and that of RANK was 1. The final score of each sample was two times the protein score of VDR plus the protein score of RANK. The ROC curve was plotted to estimate the predictive value of the final score for IFX efficacy. The results showed that the final score had an IFX effective prediction value of 0.828 (95% CI: 0.665–0.991, *p*-value = 0.013) in week 6 (**Figure 8A**), with a sensitivity of 82.4% and a specificity of 71.4%. This finding indicates that total IHC scores less than 5 for VDR and less than 7 for RANK have good predictive value for primary non-response to IFX in patients with UC. The AUC was 0.759 (95% CI: 0.565–0.953, *p*-value = 0.032) in week 14 (**Figure 8B**), with a sensitivity of 69.2% and a specificity of 72.7%.

## Discussion

Precision medicine is becoming a hot topic in the medical literature in general, with oncology studies leading the way (25, 26). The most common strategy underlying all precision medicine is that distinct patient characteristics are used to tailor the therapeutic tactics, with the help of biomarker profiles (27). Our study extracted DEGs from a publicly available database and identified several gene signatures of patients diagnosed with UC with primary non-response to IFX based on the RRA algorithm, gastrointestinal expression, and previous studies, including *CDX2*, *CHP2*, *HSD11B2*, *RANK*, *NOX4*, and *VDR*. We used the bootstrap method and an ANN analysis to confirm that the markers were repeatable for clinical application. Moreover, an independent GEO cohort was used to verify the result. We also used samples from UC patients to explore the protein expression based on the selected DEGs. The result showed a connection between the RNA and protein model, and both two models were available, but the protein model is more reliable and more conducive to clinical application. Finally, total IHC scores less than 5 for VDR and less than 7 for RANK jointly achieved an AUC of 0.828 (95% CI: 0.665–0.991, *p*-value = 0.013) in predicting PNR to IFX. The ANN analysis further confirmed these results.

UC is a chronic inflammatory disease with an increasing incidence worldwide, affecting more than 1 million individuals in Western countries and many more globally (1, 28). UC carries a life-long risk of morbidity, especially in the moderate-to-severe disease stage. Thus far, an increasing number of biologics agents have been used for UC treatment in the clinic, including IFX, vedolizumab, adalimumab, and ustekinumab. The application of biological agents benefits patients in many aspects (3, 29). Previous studies have shown that biological agents are more effective than traditional medications in terms of short-term response (OR = 4.01, 95% CI 3.08–5.23), long-term remission (OR = 2.80, 95% CI = 1.89–4.14), severe UC rescue, and colectomy rate reduction (29.2% versus 58.3%; *p* = 0.017) (21, 30–32). A meta-analysis showed that IFX was the most effective agent at inducing remission in biologic-naive patients with moderate to severe UC (33).

Nevertheless, treatment resistance remains a tremendous clinical challenge for UC patients. As the most cost-effective biologic (34), IFX shows significant curative efficacy, but close to one-third of UC patients are primary non-responders to this drug. Moreover, prior exposure to IFX may decrease the efficacy of other biologics (6, 7, 35). As IFX is most widely used in patients with moderate to severe UC, the failure of this drug as a first-line therapy could delay the onset of effective treatment. Therefore, personalized therapy for UC and predictive methods of individual response to IFX therapy are urgently needed (10). Our research responds to this pressing need and is expected to yield practical benefits in precision medicine for UC.

Six protein-coding genes predicting IFX efficacy were initially included in our study. Mostly those in the previous studies are clinical indicators, which predict IFX efficacy by responding to disease activity of UC (8, 9). Our study focuses more on predicting primary unresponsiveness than other clinical indicators and might reveal the mechanism of IFX therapeutic effects from the molecular level or pathway. Since protein expression is not always correlated with mRNA expression and protein level detection does not require fresh tissue and can avoid multiple biopsies, we used IHC to further explore the protein expression results in another dataset.

The protein-coding genes involved are strongly correlated with changes in the immune-based response and different immune cell types, including macrophages, dendritic cells (DCs), and T cells. CDX2, a transcription factor, has been shown to have a decreased expression in UC (36), play an essential role in intestinal homeostasis, and act as a context-dependent tumor suppressor in colorectal cancer. The deletion of CDX2 from the intestinal epithelium in mice leads to macrophage infiltration, causing chronic inflammatory responses (37). However, CDX2 did not revert to normal in CD patients treated with anti-TNF-α biologics (38). In our study, CDX2 did not differ between the groups by IHC. The biological function of CHP2 remains largely unknown. Guo-Dong Li et al. found that CHP2 can increase the nuclear presence of nuclear factor of activated T cells (NFATc3) and enhance activated T cell activity (39). In particular, T helper (Th) 2-mediated inflammation plays a role in UC (40). NFATs can cooperate with various transcription factors to form transcriptional complexes and integrate signaling pathways to change transcriptional patterns (41, 42). HSD11B2 and NOX4 are enriched in the hypoxia response. Tissue hypoxia, which decreases HSD11B2 and increases NOX4 expression, occurs in chronic inflammatory conditions, such as IBD. Van Welden et al. suggested that hypoxia of the colonic mucosa activates hypoxia inducible factors (HIFs) and the regulation of nuclear factor κB (NF-κB) (43). Yu et al. found that HIF-1α was upregulated in UC patients and positively related to disease progression (44). Therefore, colonic tissue hypoxia and hypoxia-induced signaling may be detection and therapeutic targets in UC (43). The reduction in HSD11B2 and the increase in NOX4 suggest a higher hypoxia response, which regulates inflammatory and immune processes and results in a complex hypoxia-immune-based microenvironment. Despite the expression of CDX2, CHP2, HSD11B2, and NOX4 related to IFX therapy and coping with inflammatory activity, their protein expression did not show a difference in the validation cohort. This finding might account for the different disease complexities and activities of UC patients between the public datasets and our enrolled subjects. We did not include these proteins in the protein prediction model.

Regarding the ultimately involved proteins, several reports from our group and others have highlighted the importance of VDR, a receptor of vitamin D, in UC. The colonic expression of VDR was inversely associated with disease activity in UC (45). Moreover, in our previous research, 25[OH]D3 levels were negatively correlated with the disease severity of UC (r = -0.371, p < 0.001) (46). A study by Shirwaikar Thomas et al. showed that in IBD patients, those with active endoscopic inflammation have a lower vitamin D level than those in remission (47). Furthermore, low pretreatment serum 25[OH]D predicted vedolizumab failure in patients with IBD (48).

UC results from T helper (Th) 2-mediated inflammation, leading to the possibility that inhibitors of Th2 cytokines might be helpful in the treatment of UC (49, 50). Vitamin D has been shown to inhibit the proliferation of T cells from patients with active UC (51), which might reduce Th2 cell-induced inflammation. Furthermore, the levels of Th2 cells were higher in anti-TNF-non-responders in UC (52). A study by Song et al. demonstrated that VDR restricts Th2-biased inflammation in the heart (53). Therefore, the reduction in VDR in colonic tissue might correlate with a strengthening of Th2-mediated inflammation and anti-TNF non-response. Bingning et al. showed that VDR activation performs a solid anti-inflammatory function in macrophages and ameliorates insulin resistance (54). VDR signaling in macrophages suppresses NF-κB activity and reduces inflammatory factor interactions (55). VDR also regulates the function of Paneth cells in releasing antimicrobial peptides to modulate the innate immune process. Thus, the regulation of VDR on immune cells might improve intestinal inflammation, leading to disease activity.

Receptor activator of nuclear factor κB (RANK), also known as TNFRSF11A, is a member of the TNF receptor superfamily. The interactions between RANK and its ligand (RANKL) regulate T cell/DC communications, DC survival, and naive T cell proliferation (56, 57). Previous studies have shown that UC is characterized by an increase in activated T cells and T-regulatory cells and a decrease in naive T-cells (58, 59). DCs monitor the surrounding microenvironment, sample antigens, and induce tolerance or incite a host defense proinflammatory response in UC (60). Therefore, a reduction in RANK might lead to an imbalance in the immune microenvironment by affecting DCs and T cells, thereby inducing UC activity.

Collectively, our study demonstrates that total IHC scores less than 5 for VDR and less than 7 for RANK were associated with non-response to IFX. The diminished expression of VDR and RANK may account for the immune-related changes in the intestinal microenvironment and reduce anti-inflammatory factors, leading to an increase in disease activity. Meanwhile, the modulation of different immune cell populations and inflammatory processes may lower anti-inflammatory cell types and weaken the immune response. Therefore, IFX may not be sufficiently robust to address this complicated inflammatory status, resulting in an inadequate therapeutic effect.

Our study has several strengths. First, we obtained transcriptome data from public datasets for the integration analysis, which is the premise of precision medicine. Second, the resampling method was used to expand the data, and then an ANN analysis was used for internal verification and prediction. We repeated the analysis process many times to show the stability of the model, forming a foundation for clinical application in the prediction of PNR. The significant proteins are readily tested in practice and are convenient for clinical application. In addition, we verified the validity of the protein profiles by IHC staining of colonic tissues from UC patients treated with IFX in our hospital. In previous studies, clinical factors, serum markers, and host genetics were demonstrated to play a role in the therapeutic response but did not accurately predict PNR. The secondary validation process in our study demonstrated that the clinical application of the immune-related signatures of primary IFX non-response in UC patients is repeatable. Furthermore, the time point of the response assessment was 6 to 8 weeks after the first IFX treatment in the GEO datasets and our enrolled subjects. A previous study showed that early measurement could better predict future remission and, thus, possibly benefit decision making (61).

Our research is not without limitations. To maintain consistency with the GEO databases, the clinical Mayo score 6–8 weeks after IFX treatment was used as the assessment when the recruited UC patients did not have endoscopy data. Thus, our study showed evidence of consistency and presented early predictive value even when an endoscopic evaluation was unavailable. However, our method may miss some patients whose endoscopic response is better or worse than their clinical response, which could increase the false-positive rate or the false-negative rate of the external verification. To reduce this bias, we also estimated the therapeutic efficacy in week 14 **(** **Figure 5B** **)**, which included an endoscopic score. The signatures also showed good predictive value, with an AUC of 0.759. Although we identified the thresholds for VDR and RANK in predicting IFX efficacy, the results showed minor differences and overlap to some extent to distinguish responders and non-responders. However, our study provides preliminary data for using proteins to predict IFX efficacy. In the future, other more sensitive protein identification methods, such as electrical detection methodologies, might be developed for the precision treatment in the clinical practice (62). Furthermore, the percentage of non-responding patients in week 14 was higher than that in week 6, indicating that early assessment is preferable as an aid for decision making. Nevertheless, large-scale prospective studies are needed to correct this limitation.

In conclusion, this study found that total IHC scores less than 5 for VDR and less than 7 for RANK were good immune-based protein signatures of PNR to anti-TNF treatment in UC patients. Applying this panel in clinical practice could help clinicians identify likely IFX non-responders before initiating therapy. Nevertheless, the practical advantage of such a tailored approach needs to be confirmed in the future.

## Data Availability Statement

The original contributions presented in the study are included in the article/**Supplementary Material**. Further inquiries can be directed to the corresponding author.

## Ethics Statement

The studies involving human participants were reviewed and approved by the Peking Union Medical College Hospital and the Chinese Academy Medical Science Ethics Committee (S-K1142). Written informed consent for participation was not required for this study in accordance with the national legislation and the institutional requirements.

## Author Contributions

XC and HY conceptualized and designed the research. XC carried out the data analysis. XC and RZ carried out the experimental procedures. HY and JQ oversaw the study and provided financial support. This manuscript was reviewed and revised by LJ, WH, XB, GR, MG, and HL. All authors contributed to the article and approved the submitted version.

## Funding

This work was funded by the National Natural Science Foundation of China (No. 81570505 and No. 81970495), the Natural Science Foundation of Beijing Municipality (No. 7202161), the Health Research & Special Projects Grant of China (No. 201002020 and No. 201502005), the Peking Union Medical College Postgraduate Innovation Fund (No. 2019-1002-71), and the National Clinical Specialty Projects along with the CAMS Innovation Fund for Medical Sciences (No. 2021-1-I2M-001).

## Conflict of Interest

The authors declare that the research was conducted in the absence of any commercial or financial relationships that could be construed as a potential conflict of interest.

## Publisher’s Note

All claims expressed in this article are solely those of the authors and do not necessarily represent those of their affiliated organizations, or those of the publisher, the editors and the reviewers. Any product that may be evaluated in this article, or claim that may be made by its manufacturer, is not guaranteed or endorsed by the publisher.
