# Supplementary material for: Artificial Neural Network Analysis-Based Immune-Related Signatures of Primary Non-Response to Infliximab in Patients With Ulcerative Colitis
Source: Front Immunol. 2021 Dec 21;12:742080. doi: 10.3389/fimmu.2021.742080 (PMC8724249; doi:10.3389/fimmu.2021.742080)
Supplement: Supplementary file 1 [file Table_1.doc]

**SUPPLEMENTARY TABLE 1 | AUC of bootstrapping and ANN analysis results of different combination of the top DEGs.**

| DEGs combination | AUC (Mean±SD) |
| --- | --- |
| All integrated DEGs | 0.872 ± 0.102 |
| Top 300 integrated DEGs | 0.857 ± 0.101 |
| Top 100 integrated DEGs | 0.855 ± 0.098 |
| Top 50 integrated DEGs | 0.847 ± 0.102 |
| Six selected DEGs | 0.850±0.103 |

**SUPPLEMENTARY TABLE 2 | AUC bootstrapping and ANN analysis results of predicting the IFX efficacy at week six and week fourteen.**

| Time point | AUC (Mean±SD) |
| --- | --- |
| Week six | 0.837 ± 0.152 |
| Week fourteen | 0.776 ± 0.162 |
